# Genome-wide association analysis reveals that EDNRB2 causes a dose-dependent loss of pigmentation in ducks

**DOI:** 10.1186/s12864-021-07719-7

**Published:** 2021-05-25

**Authors:** Yang Xi, Qian Xu, Qin Huang, Shengchao Ma, Yushi Wang, Chunchun Han, Rongping Zhang, Jiwen Wang, Hehe Liu, Liang Li

**Affiliations:** Farm Animal Genetic Resources Exploration and Innovation Key Laboratory of Sichuan Province, College of Animal Science and Technology, Sichuan Agricultural University, Chengdu, People’s Republic of China

**Keywords:** GWAS, Duck, Plumage pattern, Dose effect, EDNRB2

## Abstract

**Background:**

Birds have various plumage color patterns, and spot is a common phenotype. Herein, we conducted genome-wide association studies (GWAS) in a population of 225 ducks with different sized black spots to reveal the genetic basis of this phenomenon.

**Results:**

First, we quantified the black spot phenotype within the duck population. The results showed that the uncolored area of the body surface first appeared on the ventral side. With increasing duck age, the area of the black spots was highly conserved across the whole body surface. The GWAS results identified a 198 kb (Chr4: 10,149,651 bp to 10,348,068 bp) genetic region that was significantly associated with the black spot phenotype. The conditional GWAS and linkage disequilibrium (LD) analysis further narrowed the ultimate candidate region to 167 kb (Chr4: 10,180,939 bp to 10,348,068 bp). A key gene regulating melanoblast migration and differentiation, EDNRB2 (Endothelin B receptor-like), was found in the candidate region and having significant mRNA expression level changes in embryonic duck skin tissue with different spot sizes. The significant SNPs (single nucleotide polymorphisms) associated with the EDNRB2 gene were annotated, and two mutations (Chr4: 10,180,939 T > C and Chr4: 10,190,671 A > T) were found to result in the loss of binding sites for two trans-factors, XBP1 and cMYB. The phenotypic effect of these two mutations suggested that they can regulate the size of black spots in a dose-dependent manner, and Chr4: 10,180,939 T > C was the major allele locus.

**Conclusions:**

Our results revealed that EDNRB2 was the gene responsible for the variation in duck body surface spot size. Chr4: 10,180,939 T > C was the major allele that explained 49.5 % (dorsal side) and 32.9 % (ventral side) of the variation in duck body surface spot size, while 32.1 % (dorsal side) and 19.1 % (ventral side) of the variation could be explained by Chr4: 10,190,671 A > T. The trans-factor prediction also suggested that XBP1 and cMYB have the potential to interact with EDNRB2, providing new insights into the mechanism of action of these genes.

**Supplementary Information:**

The online version contains supplementary material available at 10.1186/s12864-021-07719-7.

## Background

Birds constitute one of the most diverse classes of animals on Earth, presenting a wide variety of body types, vocalizations, and plumage displays. Plumage color is crucial for birds, as it possesses multiple biological functions, e.g., heat retention, mate attraction, communication, camouflage, and skin protection; thus, it plays an important role in the evolution and diversity of birds [1, 2]. Additionally, in modern poultry production, plumage color is a characteristic that can be used to distinguish one breed from another and may affect consumers’ choice in some Asian countries. Revealing the genetic basis underlying plumage color is helpful for cultivating breeds that can conform to consumption demands and provides a reference for studying the origin and evolution of poultry and the protection of poultry genetic resources.

Spotted coats/plumage are a prominent color phenotype in mammals and avians and have been observed in cattle [3], donkeys [4], swamp buffalos [5], horses [6], quails [7], chickens [8] and ducks [9]. The formation of pigmentation is mainly attributed to variations in the quantity, proportion and location of eumelanin and pheomelanin, which are endogenously synthesized by melanocytes. Therefore, the distribution of pigment on the body, reflecting the absence of mature melanocytes, is caused by defects at various stages of melanocyte development, including proliferation, survival, migration, invasion of the integument, hair follicle entry and melanocyte stem cell renewal [10, 11]. According to Cieslak’s research, the spotted phenotype is mainly caused by a migration disorder of melanoblast cells. The genes responsible for melanoblast migration and melanocyte development include EDN3, EDNRB, EDNRB2, KIT and KITLG [12].

Duck (*Anas platyrhynchos*) is one of the most important domestic fowl worldwide. In China, there are 32 local domesticated breeds. Among them, the plumage color phenotypes show tremendous differences. White-spotted individuals were observed in many duck populations. Using a candidate gene study strategy, Li et al. presumed that the Arg332His mutation in EDNRB2 was associated with spotted ducks [9]. However, we observed that there were significant differences in the size of the black spots on the body surface of black-spotted ducks. Therefore, we conducted GWAS (genome-wide association studies) in duck populations with different levels of black spot sizes to determine the genetic basis of this phenomenon.

## Results

### The pigmented spots on the duck body showed a dose-dependent loss in the population

**Fig. 1:**
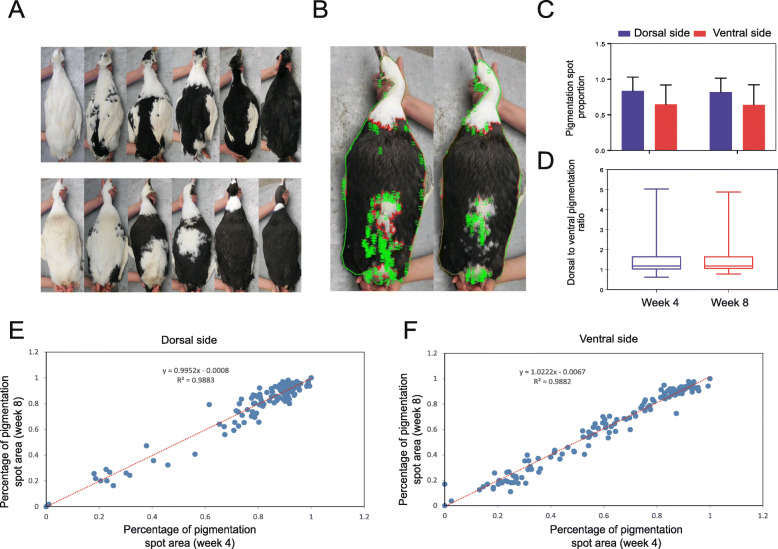
The distribution of pigmentation spots on the duck body surface.** a** The individual differences in the distribution of pigmentation spots on both the dorsal and ventral sides of ducks showed a dose-dependent, increasing trend. **b** Schematic diagram for calculating the ratio of pigmentation spots to the body surface area with IPP6 software. **c** Comparison of pigmentation spot ratios between the dorsal and ventral sides of ducks. **d** Boxplot showing the range of pigmentation spot ratio values on the dorsal side relative to the ventral side. **e & f.** Correlation analysis between the proportion of pigmentation spots at 4 weeks and the proportion at 8 weeks on ​​the dorsal (**e**) and ventral sides (**f**) of ducks

Figure 1a shows a clear individual difference in pigmentation spot distribution on both the dorsal and ventral sides of ducks (Fig. 1a). To classify the pigmentation spot on the duck body surface in more detail, we employed Image-Pro Plus 6.0 software to analyze the pigmentation spot area distributed on the dorsal and ventral sides of ducks (Fig. 1b). Overall, the pigmented spots on the dorsal side were distributed over a larger area than those on the ventral side (Fig. 1c). Figure 1d shows that 90 % of the individuals exhibited a larger pigmentation spot area on the dorsal side than on the ventral side (Fig. 1d). Combined with phenotypic observations, it is likely that the non-pigmented area first appeared on the ventral side. In this duck population, the individuals’ pigmentation area exhibited a continuously increasing trend, reflecting the dose dependence of pigmentation spot distribution. Additionally, we performed a correlation analysis between the percentage of the pigmented area in 4-week-old and 8-week-old ducks. For both the dorsal and ventral sides, there were high positive correlations with an R^2^ greater than 0.98, indicating that the size of the pigmentation spot was conserved on the duck body surface, even as the age of the duck increased (Fig. 1e f).

### Population structure and SNP distribution on chromosomes

**Fig. 2:**
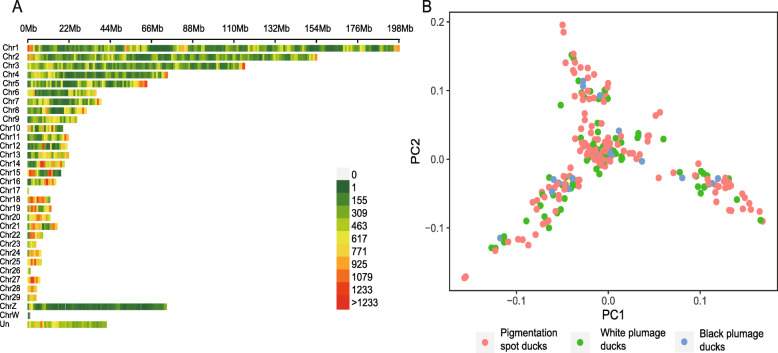
SNP information and population structure analysis.** a** The number of SNPs within a 1 Mb window size distributed across all chromosomes. **b** Principal Component Analysis. PCA was conducted on the SNP information of each sample using the GCTA tool. The red, green and blue dots represent individuals with different plumage patterns

 For all 225 ducks, a total of 184, 792 SNPs with a minor allele frequency (MAF) > 0.03, a major allele frequency < 0.99 and a maximum missing rate < 0.1 were finally obtained and used for the subsequent analysis. All filtered SNPs with an average density of 168 SNPs/Mb were distributed on 29 autosomal chromosomes, ChrZ, ChrW and Un (unplaced scaffolds) [13] (Fig. 2a). Principal component analysis (PCA) was conducted by GCTA software to analyze the population structure of all 225 ducks. Three potential subpopulations were found, indicating that population stratification existed in our genomic samples, but it had little effect on the plumage pattern phenotype. Individuals with different plumage patterns were evenly distributed in the three clusters (Fig. 2b).

### GWAS showed a candidate region on chromosome 4

**Fig. 3:**
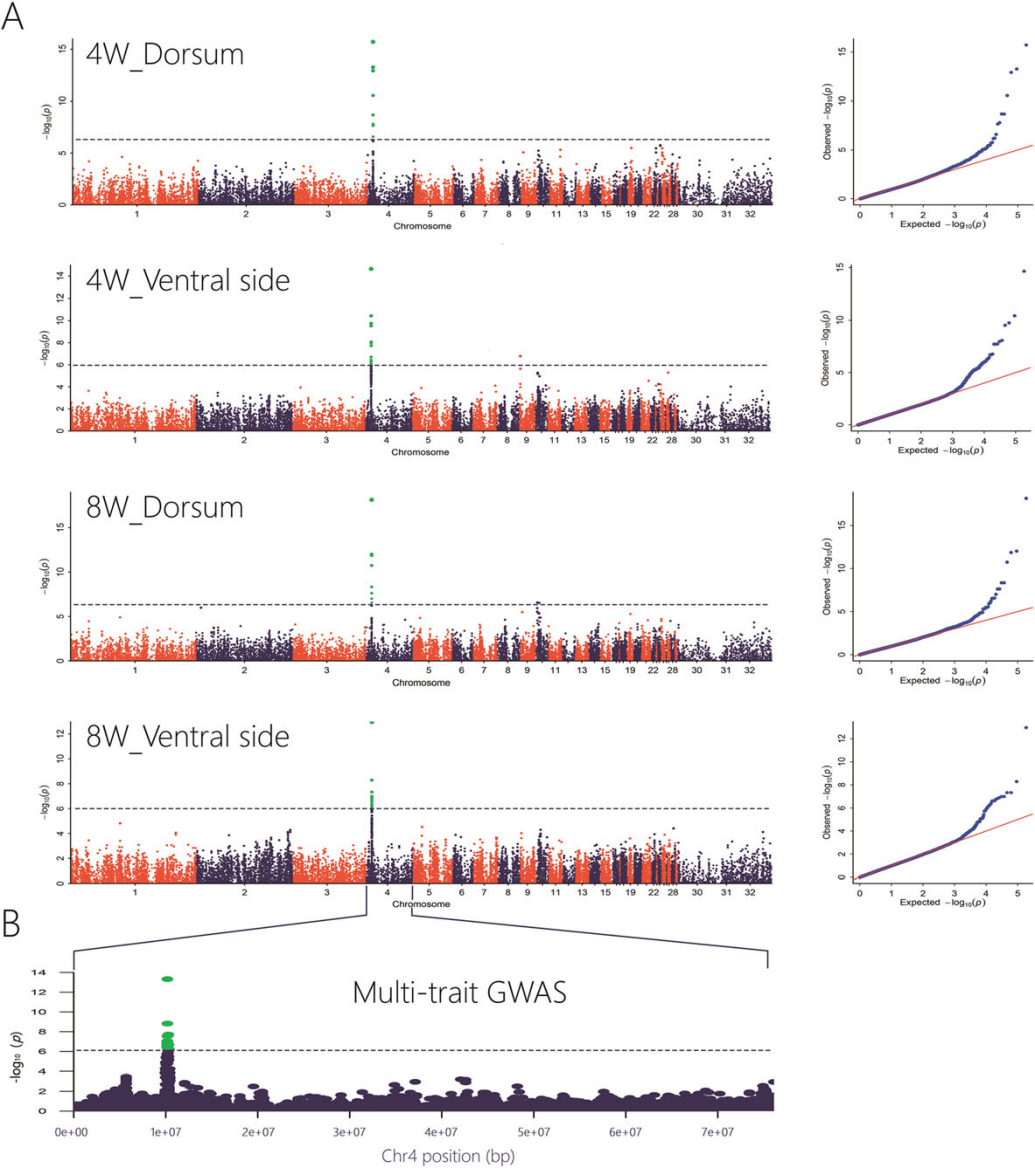
Genome-wide association studies between genotypes and pigmentation phenotypes.** a** Single-trait GWAS analysis. Manhattan (left) and QQ plots (right). **b** The consensus candidate region was identified on chromosome 4 by multi-trait GWAS. The gray line represents the Bonferroni corrected significance threshold (-log_10_*P* = 6). The x-axis shows the physical positions of each marker along the chromosome, and the y-axis shows the - log_10_*P* values of SNPs

We performed GWAS based on the genotypes and the pigmented area ratios of 4 comparison groups (4 W-dorsal side 4 W-ventral side, 8 W-dorsal side and 8 W-ventral side). A significant signal peak was observed at the same position on chromosome 4 among all groups (Fig. 3a). The SNPs with significance (-Log_10_*P* > 6) were distributed in an ~ 370 kb region from 9,981,595 bp to 10,348,068 bp on chromosome 4 (4 W-dorsal side: 10,180,939 bp – 10,346,153 bp; 4 W-ventral side: 9,981,595 bp − 10,348,068 bp; 8 W-dorsal side: 10,180,939 bp – 10,346,153 bp; and 8 W-ventral side: 10,149,651 bp – 10,346,153 bp) (Table S1). The signals for all four pigmentation spot distributions showed a complete overlap with each other, implying that a consensus genetic foundation may modulate the spatial-temporal distribution of pigmentation spots. To determine a more accurate candidate region, we conducted a multi-trait integrated GWAS analysis by mvLMM (multivariate linear mixed model). Based on this analysis, some of the significant SNPs disappeared, and we identified a 198 kb consensus candidate region (Ch4: 10,149,651 bp – 10,348,068 bp) on chromosome 4 (Fig. 3b & Table S1). The candidate region harbored 11 genes, of which 6 were protein coding genes and 5 were pseudogenes (Table S2). The protein-coding genes were LOC101789516, VAMP7, LOC101794354, LOC101805249, LOC101805050 and SPRY3. The most significant SNP (Chr4: 10,180,939) was in the promoter region of LOC101805249, which encodes an endothelin B receptor-like protein and is also known as EDNRB2.

### EDNRB2 was identified as the causative gene affecting black spot size

**Fig. 4:**
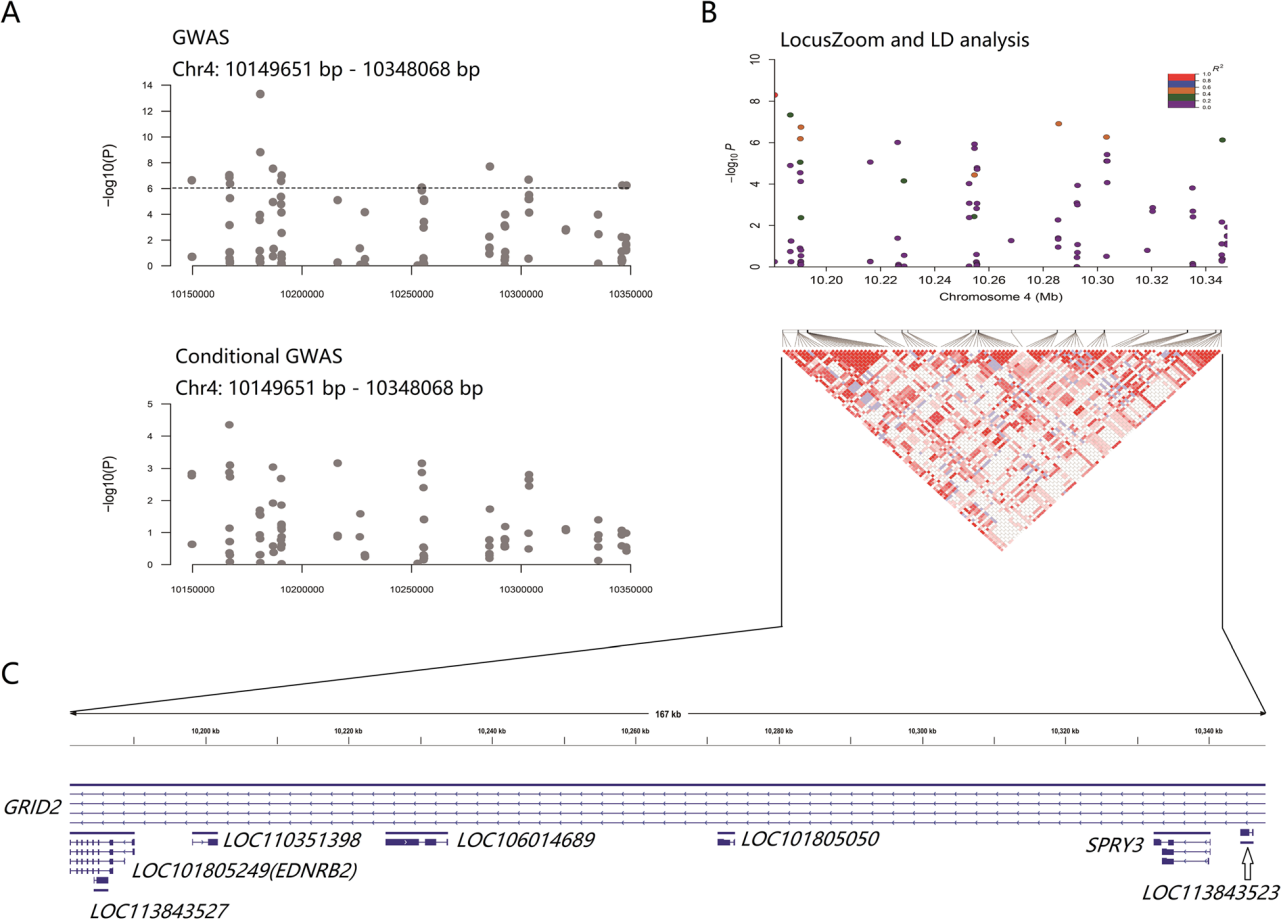
Candidate region screening. The x- and y-axes represent the specific chromosome position and –log_10_P value, respectively. **a** Comparison between the GWAS on chromosome 4 and conditional GWAS after removing the effect of the most significant SNP. After integrating the genotype of Chr. 4: 10,180,939 bp into the analysis as a covariate, all of the remaining SNPs lost significance. **b** LD analysis in the region with significant SNPs. R^2^ > 0.2 was considered to indicate linkage. A ~ 167 kb LD block was confirmed, and there were also several independent small-range LD blocks within this region. **c** Genes within the 167 kb LD block

To further screen the candidate region and potential causative genes, we performed conditional GWAS by removing the effect of the most significant SNP (Chr4: 10,180,939 bp) from the multi-GWAS analysis. After removing the effect of the most significant SNP, the remaining significant SNPs almost dropped below the threshold line, implying that there was potential linkage disequilibrium (LD) among SNPs in this region (Fig. 4a; Table S3). Therefore, LD analysis in the 198 kb candidate region was conducted. R^2^ > 0.2 was considered to indicate a linked correlation between the most significant SNP and each remaining SNP. The LD block was confirmed to span an ~ 167 kb region from 10,180,939 bp to 10,348,068 bp covering 8 genes: GRID2, LOC101805249, LOC113843527, LOC110351398, LOC106014689, LOC101805050, SPRY3 and LOC113843523 (Fig. 4b, Table S4). Further annotation revealed that LOC113843527, LOC110351398, LOC106014689 and LOC113843523 were pseudogenes, while GRID2 (glutamate ionotropic receptor delta type subunit 2), LOC101805249 (endothelin B receptor-like; also known as EDNRB2), LOC101805050 (brain-specific homeobox/POU domain protein 3) and SPRY3 (sprouty RTK signaling antagonist 3) were coding genes (Fig. 4c & Table S5). SNP annotation showed that only two coding genes were identified as having relationships with significant SNPs in the candidate region (Table S6). Among them, EDNRB2 was previously indicated to affect the proliferation and migration of melanocytes.

### Phenotypic effect of significant SNPs related to EDNRB2

**Fig. 5:**
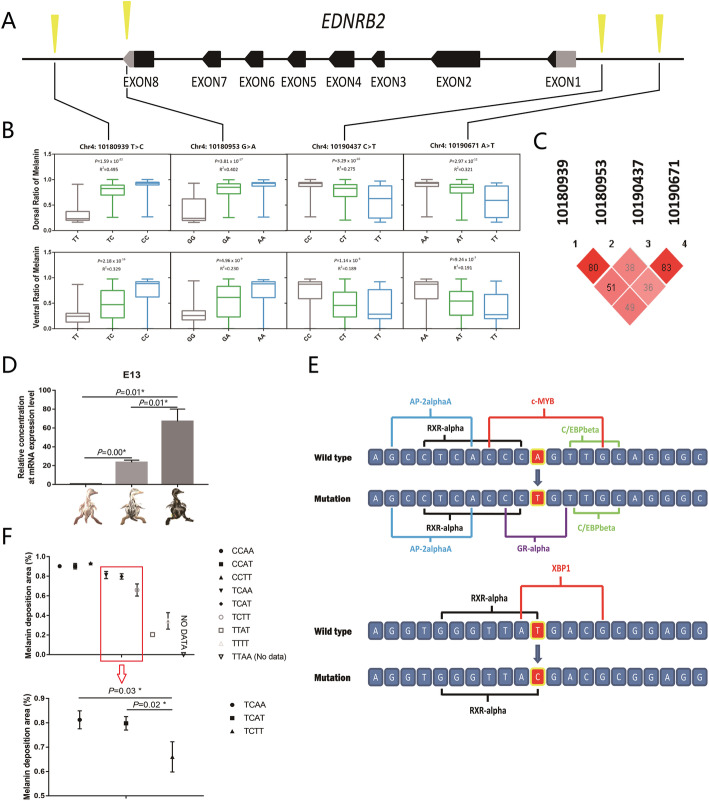
Phenotypic effect of single SNPs and their combination.** a** The blocks show the exons of EDNRB2. The arrow indicates the direction of gene transcription. The cones represent significant SNPs associated with the EDNRB2 gene. **b** The phenotypic effect of every single significant SNP was analyzed on the dorsal and ventral sides. *P* values and R^2^ values were calculated using a general linear model (GLM). *P* values less than 0.05 were considered significant. **c** Relatively high LD was found among the 4 SNPs. The numbers in the diamond represent R^2^ values. **d** The FPKM (fragments per kilobase of transcript per million mapped reads) values of the EDNRB2 gene in duck embryos with different melanin deposition areas were calculated based on our previous RNA-seq data [14]. The expression of EDNRB2 showed a significant increasing trend with increasing melanin area. **e** Changes in the binding sites of Chr4: 10,180,939 T > C and Chr4: 10,190,671 A > T were demonstrated. The sequence used in the analysis was 10 bp of the left and right flanks of the mutation site. **f** The genotypes were identified using Chr4: 10,180,939 and Chr4: 10,190,671. E.g. When Chr4: 10,180,939 had the C/C allele and Chr4: 10,190,671 had the A/A allele, it would be shown as CCAA. The *P* values were calculated by *T*-test, and values less than 0.05 were considered significant

The SNP annotation result identified the specific positions of the significant SNPs in the candidate region. Among them, 4 SNPs (Chr. 4: 10,180,939 T > C; Chr. 4: 10,180,953 G > A; Chr. 4: 10,190,437 C > T; and Chr. 4: 10,190,671 A > T) were found to have a relationship with the EDNRB2 gene (Fig. 5a, Table S6). Therefore, we investigated the phenotypic effects of different genotypes according to these 4 SNPs. The results showed that these SNPs were all significantly associated with the size of black spots (*P* < 0.05). Moreover, the phenotypic values of different genotypes exhibited gradual increasing or decreasing trends (Fig. 5b). Then, we conducted LD and haplotype analysis on these 4 SNPs. Relatively high LD relationships were found (Fig. 5c), and 8 haplotypes were identified (H1-H8) (Table S7). After genotyping the haplotypes and performing correlation analysis (the groups with < 3 individuals were excluded), we found that the combination of these 4 alleles could explain 52.7 % of the variation in dorsal spot size (Table S7). Although the phenotypic effects of H7H1 and H1H1 were stronger than those of the other haplotypes, there were few individuals carrying the H7 haplotype and no H7 homozygous individuals, which impeded recognition of the superior haplotype. However, considering the phenotypic effect of a single mutation, we speculated that H1 (CACA) was more likely to be the superior haplotype.

**Fig. 6:**
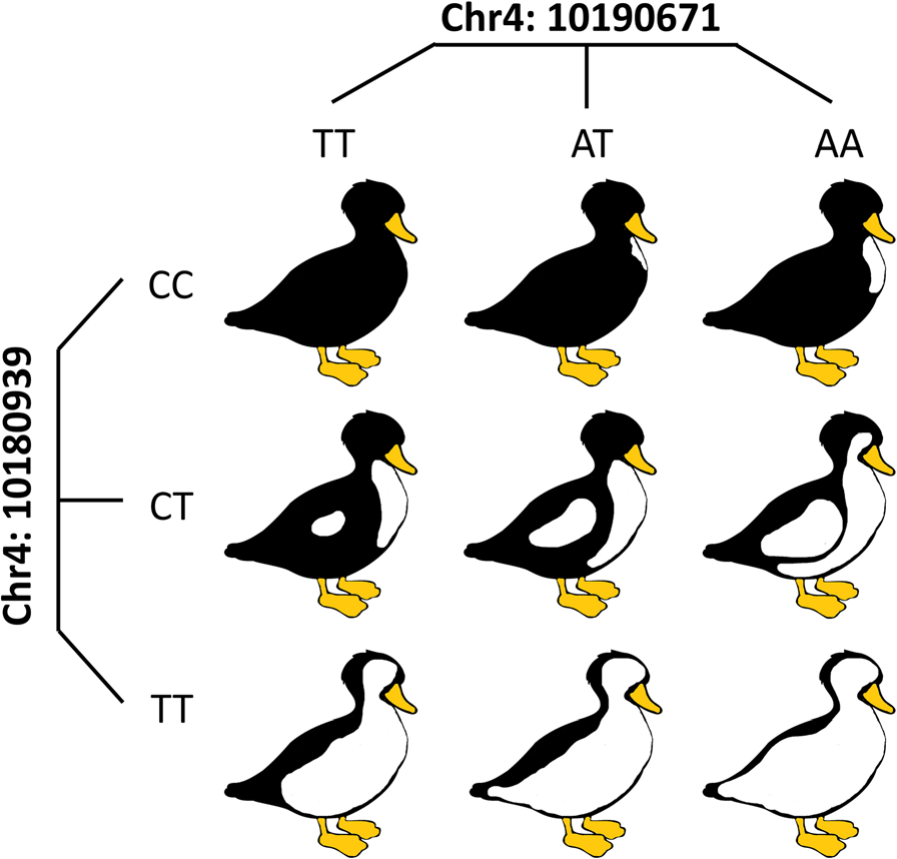
Sketch map of the phenotypic dose effect of the alleles at two SNPs. Mutation in the major allele (Chr4: 10,180,939 T > C) could lead to large-scale loss of pigment deposition, and that in the minor allele (Chr4: 10,190,671 A > T) had the ability to subtly regulate the phenotype

To investigate the relationship between the phenotype and EDNRB2 mRNA expression level, we examined the RNA-seq results of our previous study about the pigment deposition process during the embryonic period [14]. The results showed that the expression level of EDNRB2 increased with increasing melanic areas on the body surface of duck embryos (Fig. 5d). To determine whether there were any trans-factor binding site changes, the 20-bp sequence around each mutation was extracted for transcription factor binding site prediction. The results showed that 2 potential trans-acting factor binding sites for XBP1 (X-box binding protein 1) and cMYB (v-myb avian myeloblastosis viral oncogene homolog) disappeared (Chr. 4: 10,180,939 T > C corresponded to XBP1 and Chr. 4: 10,190,671 A > T corresponded to cMYB) (Fig. 5e, Table S8). The combination analysis of the two alleles showed a significant dose effect on the phenotype, and Chr4: 10,180,939 T > C was likely to be the major mutation controlling the size of the body surface melanin deposition area (Fig. 5f). Phenotypically, the mutation inhibited melanin deposition. Then, we explored the fine regulation of Chr. 4: 10,190,671 A > T when Chr. 4: 10,180,939 T > C was fixed as the T/C genotype; under this situation, the number of samples of each genotype in the combination group was more than 10, which resulted in better statistical significance. The results showed that Chr. 4: 10,190,671 A > T could increase the melanin deposition area to a small extent (Fig. 5f). Based on the above results, we constructed a dose effect model of the phenotypes resulting from the two mutations (Fig. 6).

## Discussion

The spotted phenotype is a common plumage variant in avians. Through our phenotypic analysis, we found that the size of black spots on the body surface of ducks varied greatly. The size of the black spots on ducks hardly changed during adulthood (comparison between 4-week-old and 8-week-old ducks). The genetic basis of the spotted plumage pattern in ducks has been previously identified. The c.995 G > A mutation in EDNRB2 was significantly associated with a spotted plumage pattern in ducks [9]. However, a single SNP in the coding region of the gene has limitations regarding the complex and highly variable phenotype. Therefore, we performed GWAS based on the phenotypic value of duck black spot size. Two coding genes on Chr. 4, GRID2 and EDNRB2, were identified after GWAS and fine mapping analysis. The GRID2 gene is mainly expressed in the cerebellum [15, 16]. At present, GRID2 is mainly considered to play an important role in maintaining cerebellar function. The loss of function of this gene led to cerebellar ataxia and many complications in humans and mice [17–19]. However, the gene has not been found to affect the process of pigment deposition. EDNRB2 is a homolog of EDNRB; it belongs to the endothelin receptor (EDNR) gene family and has been lost in mammalian lineages [20]. Our study observed that the formation of non-pigmented areas originated from the ventral side. This phenomenon was consistent with the migration and localization process of NCCs (neural crest cells), which have the potential to differentiate into melanoblasts. NCCs migrate from the dorsal to ventral sides via the dorsolateral pathway during the embryonic stage [21], and the expression of EDNRB2 plays an essential role in this process [22, 23]. The RNA-seq results also showed that the expression of EDNRB2 in duck embryos with different degrees of black spots was significantly different. Therefore, we speculated that the reason for the white spots originating from the ventral side was a migration disorder of NCCs mediated by EDNRB2 expression changes. Our previous study revealed that a 14-bp insertion in exon 3 of EDNRB2 led to white plumage color in Chinese geese, while heterozygous individuals showed a spotted phenotype [24]. In summary, we believe that EDNRB2 is the gene responsible for controlling body surface black spot size.

The shape and size of pigmented areas are related to the distribution of pigment in the body. Studies have shown that color patterns are regulated by pigmentation genes in a dose-dependent manner by modulating pigment production [25]. The South African Boer goat displays a characteristic white-spotted phenotype, in which pigment is limited to the head. Menzi et al. reported that this white-spotted phenotype may be controlled by the EDNRA locus, another member of the EDNR family, in a dose-dependent manner [26]. Our results supported a similar regulation mode. Chr. 4: 10,180,939 T > C was identified as the major mutation that could explain 49.5 % of the variation in duck body surface spot size on the dorsal side and 32.9 % on the ventral side. Moreover, this mutation was the most significant SNP in the GWAS results. Another important mutation, Chr. 4: 10,190,671 A > T, could regulate the spot size phenotype more subtly.

Notably, the 2 predicted trans-factors, XBP1 and cMYB, were both reported to be associated with pigmentation or pigment-related diseases. XBP1 is an important candidate gene for vitiligo. Ren et al. demonstrated that the modification of XBP1 expression at the transcriptional level by a germline regulatory polymorphism has an impact on the development of vitiligo [27]. cMYB was also proven to have the ability to promote melanocyte differentiation and possibly proliferation [28]. These studies showed that XBP1 and cMYB can inhibit and enhance melanin synthesis, respectively. This result was also consistent with our experimental results. In the analysis of the phenotypic effect of a single locus, Chr4: 10,180,939 T > C (XBP1 binding site) and Chr4: 10,190,671 A > T (cMYB binding site) inhibited and increased the size of black spots, respectively, after mutation. The EDNRB signaling pathway has been considered a potential therapeutic target to promote repigmentation in hypopigmentation disorders [29]. Our findings provide novel insights into the functional mechanism of these genes. Exploring the interaction of the EDNR family with cMYB and XBP1 may help to better understand the pathogenesis of vitiligo and identify treatments.

## Conclusions

The present study further explained the genetic mechanism of the complex shape of the pigmented area on the duck body surface. Our results revealed that EDNRB2 was the gene responsible for the duck body surface spot size variation. Chr4: 10,180,939 T > C was the major allele that explained 49.5 % (dorsal side) and 32.9 % (ventral side) of the variation in duck body surface spot size, while 32.1 % (dorsal side) and 19.1 % (ventral side) of the variation could be explained by Chr4: 10,190,671 A > T. The combination of two genetic loci can regulate the size of black spots in a dose-dependent manner. The trans-factor prediction also suggested that XBP1 and cMYB potentially interact with EDNRB2, providing a novel insight into the mechanism of action of these genes.

## Materials and methods

### Animals and sampling

 A total of 300 ducks from the intercross population resulting from a cross between the GF2 strain (a hybrid strain constructed by SICAU) and Jianchang duck (a local breed in Sichuan, China) were raised on a net floor at the poultry farm of Sichuan Agricultural University. GF2 and Jianchang duck both have inconspicuous and uneven body surface pigmentation; this variation was amplified in the F1 generation. The variation in spot sizes shown in Fig. 1 A appeared in the F1 generation and was stable in the F2 and F3 generations. In view of the fact that the F3 generation showed relatively sufficient phenotype segregation, we believe that this population was the ideal material to study the genetic mechanism of spot size variation. All ducks used in the present study were kept under identical environmental and management conditions. All individuals had free access to food and water.

### Phenotype collection

Pigmentation spots on the body surface of all ducks were photographed under the same conditions. First, the digital camera was set to manual exposure, and each image was collected under identical exposure conditions, including exposure time and aperture. Then, the obtained images were imported into IPP 6.0 software (Media Cybernetics, USA) and magnified by the same multiplication factor. Using an irregular shape tool incorporated in the software, the area of interest (AOI) of each pigmentation spot was selected, and the geometric size of each region was measured. Pigmentation spot areas on the dorsal and ventral sides of ducks were measured. The proportion of pigmentation distribution on the body surface for each duck was calculated. Three replicates were made for each measurement, and the average values were taken as the final phenotype. A total of 300 ducks were selected to measure the pigmentation phenotypes at 4 and 8 weeks old.

### SLAF (specific-locus amplified fragment) library preparation for sequencing

 Blood samples were collected from the veins under the wings of ducks. A total of 225 blood samples of pigmented ducks were selected for DNA extraction using the phenol-chloroform protocol. The quality and quantity of DNA were examined by Nanodrop and agarose gel electrophoresis [30].

Our study relied on SLAF to generate the genotypes of all ducks. Thus, the duck genome was selected as the reference genome for electronic enzyme digestion prediction. A single-nucleotide (A) overhang was added to the digested fragments using the Klenow fragment (3’→5’ exonuclease and 5’→3’ polymerase) (NEB) and dATP at 37 ℃. Next, polyacrylamide gel electrophoresis (PAGE)-purified duplex tag-labeled sequencing adapters (Life Technologies) were ligated to the A-tailed fragments using T4 DNA ligase. Polymerase chain reaction (PCR) using diluted restriction-ligation DNA samples, dNTPs, Q5® High-Fidelity DNA Polymerase and PAGE-purified PCR primers AATGATACGGCGACCACCGA and CAAGCAGAAGACGGCATACG (Life Technologies) was performed. PCR products were purified using Agencourt AMPure XP beads (Beckman Coulter, High Wycombe, UK) and pooled. Pooled samples were separated by electrophoresis using a 2 % agarose gel; 300-500-bp fragments (including indices and adaptors) were excised and purified using the QIAquick Gel Extraction Kit (Qiagen). Gel-purified products were sequenced on the Illumina HiSeq 2500 system (Illumina, Inc., San Diego, CA, USA) per the manufacturer’s directions [31]. Reads with a single-base sequencing error rate over 1/100 were discarded.

### Mapping and genotyping of reads

The SLAF clean reads were mapped to the duck reference genome (GCA_002224895.1) with Burrows-Wheeler alignment (BWA aln) [32] using the default parameters. SNP calling was performed exclusively using GATK (version 3.5) [33], and the output was further filtered using VCFtools (version 0.1.15) [34]. SNPs were filtered based on the following criteria: (1) SNPs had to have a minor allele frequency > 0.05 and a major allele frequency < 0.99; (2) the maximum missing rate was < 0.1; and (3) SNPs could only have two alleles.

### PCA

PCA was performed based on all SNPs using the GCTA tool [35]. This software applies PCA to genetic data to analyze the population structure. Three potential subpopulations were clearly identified. The figures were then plotted using the first and second principal components with R packages.

### GWAS

Population structure and cryptic relationships were considered to minimize false positives and increase statistical power. A mixed linear model program emmax was used for the association analysis [36]. The first three principal component values (PCA eigenvectors) derived from whole genome SNPs were set as a fixed effect in the mixed model to correct for population stratification [37]. The random effect was the kinship matrix estimated across all individual whole-genome SNPs. A quantile-quantile (QQ) plot was drawn to detect false positives due to population stratification. In the QQ plot, the ordinate was the observed SNP P-value, and the abscissa was the theoretical P-value determined using a chi-squared distribution. Conditional association analysis was carried out using a similar process while fitting the most significant SNP Chr4:10,180,939 as a covariate. In addition, we used the mvLMM (multivariate linear mixed model) in GEMMA software to carry out multiple trait integrated GWAS analysis [38]. All Manhattan diagrams were made by R (v3.5.1) using the package ‘qqman’ [39].

### LD and haplotype analysis

VCFtools was used to retrieve individual genotypes in the region of interest. LD analysis between the most significant SNPs and other SNPs in the candidate region was conducted by Plink, and a Locuszoom graph was generated by R (v3.5.1) [40]. Haploview software was used to analyze the total LD in candidate regions and the haplotypes of four candidate SNPs [41]. The trait involved in this study was quantitative. Therefore, we used a general linear model to analyze the association between haplotype and phenotype.

### Gene expression analysis

The duck embryo models with three levels of spot size variation were from Peking duck (non-pigmented), GF2 duck (moderately pigmented) and Heiwu duck (extensively pigmented). The EDNRB2 expression level in samples with different spot sizes was analyzed using our previous RNA-seq data [14]. Considering that pigment deposition was almost complete in the embryonic stage and that the phenotypes of the samples in our previous study were basically similar to what we used in the present study, the model should be reliable for studying the expression changes of EDNRB2. Low-quality reads were filtered out using stringent criteria by FASTX (v0.0.13), including (1) reads with more than 50 % of bases with quality < 20; (2) reads with base quality < 10 at the 3’ end; (3) reads with overrepresented adaptors; (4) reads having an ‘N’ base; and (5) reads shorter than 20 bp. Hisat2 (v2.1.0) was used to align the clean data to the reference genome of Peking duck (IASCAAS_PekingDuck_PBH1.5). The mapped data were collated and formatted by Samtools. Gffcompare was used to check the assembly of transcripts [42]. Then, Stringtie was used to calculate the gene expression level [43].

### Trans-factor prediction

The online software PROMO (http://alggen.lsi.upc.es) was used to predict whether there were any trans-acting factor changes caused by the 4 SNPs. The sequence used in the analysis was 10 bp of the left and right flanks of the mutation site. We constructed the sequence template before and after mutation. To increase the accuracy of prediction, the maximum matrix dissimilarity rate value was reduced to 4.

### Statically analysis

The *P*-values from the comparison and correlation analysis between the phenotypic value and genotype were analyzed by T-test and a general linear model with the SPSS 19.0 software package. Differences at *P* < 0.05 were considered significant.

## Supplementary Information

**Additional file 1: Table S1. **Top 2000 SNPs from GWAS and multi-trait GWASresults. **Table S2. **Gene annotationin 198kb genomic region. **Table S3. **ConditionalGWAS result of candidate region. **TableS4.** Linkage disequilibrium analysis between the leader SNP and the others. **Table S5. **Gene annotation in theultimate candidate region on Chr4. **TableS6.** Significant SNP annotation in the ultimate candidate region on Chr4. **Table S7. **Haplotype and correlationanalysis. **Table S8. **The predictionof trans-factor binding sites based on the significant SNPs related to EDNRB2.

## Data Availability

The Genome sequencing raw data was deposited in NCBI’s SRA database (https://trace.ncbi.nlm.nih.gov/Traces/sra/sra.cgi?view=studies&f=study&term=&go=Go; Accession: SRP314426). The RNA sequencing raw data was previously released in the GSA database (https://bigd.big.ac.cn/gsa/browse/browse; Accession: CRA003266).

## Abbreviations

GWAS: Genome-wide association studies
LD: Linkage disequilibrium
SNP: Single nucleotide polymorphism
MAF: Minor allele frequency
PCA: Principal component analysis.
GRID2: Glutamate ionotropic receptor delta type subunit 2.
EDNRB2: Endothelin B receptor-like
SPRY3: Sprouty RTK signaling antagonist 3
XBP1: X-box binding protein 1
cMYB: v-myb avian myeloblastosis viral oncogene homolog
FPKM: Fragments Per Kilobase of exon model per Million mapped fragments
EDNR: Endothelin receptors
NCC: Neural crest cell
GLM: General Linear Model
AOI: Area of interest
SLAF: Specific-Locus Amplified Fragment
